# Characterization of the complete mitochondrial genome of *Notonecta amplifica* Kiritshenko, 1930 (Hemiptera: Notonectidae) and its phylogenetic implications

**DOI:** 10.1080/23802359.2021.2008831

**Published:** 2021-12-15

**Authors:** Min Li, Ting Lei, Guobin Wang, Luming Wang

**Affiliations:** a Department of Biology, Taiyuan Normal University, Jinzhong, China; b College of Plant Protection, Shanxi Agricultural University, Taigu, China

**Keywords:** *Notonecta amplifica* Kiritshenko 1930, complete mitochondrial genome, phylogeny

## Abstract

The complete mitochondrial genome of *Notonecta amplifica* Kiritshenko, 1930 (Hemiptera: Notonectidae) was sequenced by high-throughput sequencing (HTS). The whole length is 15,150 bp, including 13 protein-coding genes (PCGs), 22 transfer RNA (tRNAs), two ribosomal RNA (rRNAs) and a 528 bp control region (D-loop). The nucleotide is with a base composition of A (43.01%), G (10.32%), C (13.87%) and T (32.80%). The total length of the coding sequences encoding 3,694 amino acids is 11,113 bp, accounting for 73.35%. The lengths of 12S rRNA and 16S rRNA are 800 and 1,251 bp, respectively. The Bayesian phylogenetic analysis of 20 taxa indicate that *N. amplifica* is closely related to *Notonecta montandoni*, with posterior probability value of 1.00. The complete mitochondrial genome sequence would provide useful genetic information for future taxonomic and phylogenetic classification of *N. amplifica*.

*Notonecta amplifica* Kiritshenko, 1930 (Hemiptera, Notonectidae) has the longest body length in the family Notonectidae (Hungerford 1933), which are important invertebrate predators and natural enemies of mosquitoes (Shaalan and Canyon 2009; Dalal et al. 2019; Dambach 2020). Up to now, genomic resources for *N. amplifica* are blank. The elucidation of their complete mitogenome will enhance our current knowledge of the structure and organization of mitogenomes in *Notonecta*. Hence, we sequenced, *de novo* assembled and annotated the complete mitochondrial genome of *N. amplifica*.

The *N. amplifica* specimens were collected from Wuying River (48°2′57.12″N, 129°16′51.80″E), Fenglin County, Yichun City, Heilongjiang Province, China, on 27 August 2016. Voucher species were deposited in the Insect Molecular Systematics Laboratory, Department of Biology, Taiyuan Normal University, (Jinzhong, China) under the collection number 2018NAHLJHY1 (contact Dr. Min Li, Email: limin12nk@163.com). The complete genome sequence was deposited in GenBank under accession number MZ305077.

The complete mitochondrial genome of *N. amplifica* has a length of 15,150 bp, and encodes 37 genes, including 13 PCGs, 22 tRNAs and two rRNAs. All genes are distributed on two strands. 14 genes including trnQ-Gln, trnC-Cys, trnY-Tyr, trnF-Phe, *ND5*, trnH-His, *ND4*, *ND4L*, trnP-Pro, *ND1*, trnL-Leu (CUN), 16S rRNA, trnV-Val and 12S rRNA are located on the minor coding strand (N-strand), while the other 23 genes are on the major coding strand (J-strand). The D-loop region is considered as misc_feature. The nucleotide composition of *N. amplifica* mitochondrial genome has an asymmetric nucleotide composition (43.01% A, 13.87% C, 10.32% G and 32.80% T). The percentage of G + C is 24.19%, and the percentage of A + T is 75.81%, which is similar to the backswimmer, *Notonecta montandoni* (24.27% of G + C and 75.73% of A + T) (Li et al. 2019).

Of the 13 PCGs, 12 genes share the start codon ATN (three with ATA; one with ATT; eight with ATG), while *COI* starts with TTG. Nine PCGs terminate with a complete termination codon (seven with TAA, two with TAG), and four genes terminate with T. The length of the 13 PCGs is 11, 113 bp. The 22 tRNAs range from 62 to 72 bp, of which 14 tRNA genes are encoded by J-strand and the other eight are encoded by N-strand. The two rRNAs were 800 bp (12S rRNA) and 1251 bp (16S rRNA) long and are separated from each other by trnV-Val. The D-loop region is located between 12S rRNA and trnI-Ile with a total length of 528 bp, which accounts for 3.49% of the total length.

Bayesian inference of likelihood was implemented in MrBayes 3.2.3 (Ronquist et al. 2012) for the PCGs data set. In total, 13 PCGs of 20 species (17 notonectid taxa and three outgroups) were extracted to construct the Bayesian phylogenetic tree (Figure 1). The monophyly of each of six superfamilies in Nepomorpha is well supported (Li et al. 2012) and Notonectoidea is proved a sister group to Pleoidea (Hebsgaard et al. 2004). The *N. amplifica* is closely related to *N. montandoni* with posterior probability value of 1.00. And then they form successive sister group with *Enithares tibialis* in Notonectoidea. The complete mitochondrial genome sequence of *N. amplifica* will enrich the genome data of Nepomorpha, and will be useful for taxonomy research, phylogeny and biogeography of this group.

**Figure 1.**
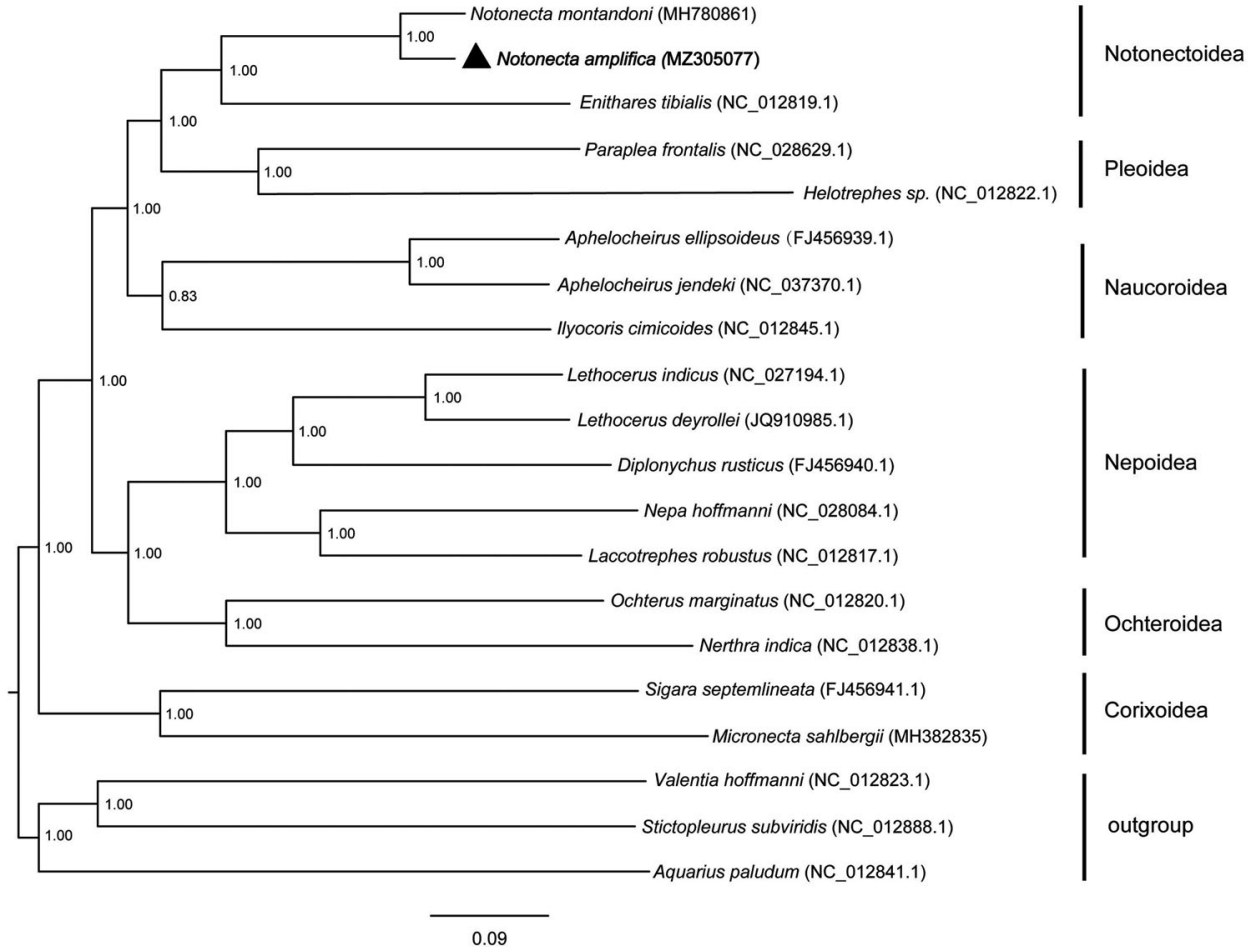
Phylogeny estimation based on 13 PCGs. The species name is followed by GenBank accession numbers. Bootstrap support values (50%) are indicated at each node.

## Data Availability

The mitochondrial genome sequence data that supports the findings of this study for *N. amplifica* are openly available in GenBank of NCBI at https://www.ncbi.nlm.nih.gov/nuccore under the accession number MZ305077. The associated BioProject, SRA data and BioSample accession numbers are PRJNA751698, SRR15329827 and SAMN20524653, respectively.
